# Adaptive changes of coral *Galaxea fascicularis* holobiont in response to nearshore stress

**DOI:** 10.3389/fmicb.2022.1052776

**Published:** 2022-11-08

**Authors:** Wentao Zhu, Ming Zhu, Xiangbo Liu, Jingquan Xia, Hao Wang, Rouwen Chen, Xiubao Li

**Affiliations:** ^1^College of Ecology and Environment, Hainan University, Haikou, China; ^2^State Key Laboratory of Marine Resource Utilization in South China Sea, Hainan University, Haikou, China; ^3^College of Marine Science, Hainan University, Haikou, China

**Keywords:** multiple stressors, coral holobiont flexibility, physiological plasticity, microbial reorganization, interaction network

## Abstract

Global change and local stressors are simultaneously affecting the nearshore corals, and microbiome flexibility may assist corals in thriving under such multiple stressors. Here, we investigated the effects of various environmental variables on *Galaxea fascicularis* holobiont from nearshore and offshore reefs. These nearshore reefs were more turbid, eutrophic, and warm than offshore reefs. However, coral physiological parameters did not differ significantly. Corals under stressful nearshore environments had low symbiont diversity and selected more tolerant Symbiodiniaceae. The bacterial diversity of offshore corals was significantly higher, and their community composition varied obviously. Diffusion limitations and environmental heterogeneity were essential in structuring microbial communities. Functional annotation analysis demonstrated significant differences between nearshore and offshore corals in bacterial functional groups. Environmental stress significantly reduced the complexity and connectivity of bacterial networks, and the abundances of keystone taxa altered considerably. These results indicated that corals could thrive nearshore through holobiont plasticity to cope with multiple environmental stresses.

## Introduction

Reef-building stony corals are the basis for the formation of coral reef ecosystems, often referred to as “holobionts” consisting of animal hosts, Symbiodiniaceae, as well as other microbial partners, including bacteria, archaea, fungi, and viruses (Rosenberg et al., 2007; Peixoto et al., 2017). Collaborative relationships between various partners ensure the health of holobiont under different environmental conditions, and research shows that intact coral microbial communities are critical for coral immunity and health (Reshef et al., 2006; Rosenberg et al., 2007). In addition, the nutrient exchanges between corals and their algal symbionts enable coral reefs to survive in oligotrophic waters, and the autotrophic photosynthesis of Symbiodiniaceae provides most of the daily metabolic energy requirements for coral hosts (LaJeunesse et al., 2018). Corals often host different Symbiodiniaceae in response to changes in available resources, which can confer resilience of their host to environmental instability, leading to a more advantageous state, critical for supporting coral survival (Röthig et al., 2020; Ros et al., 2021). Other coral-associated microbes are widely studied, as they have also been shown to provide essential contributions to coral holobiont functioning (Reshef et al., 2006; Rosenberg et al., 2007). Furthermore, coral-associated bacterial communities also significantly enhance coral holobiont’s environmental resilience, which is greatly influenced by different environmental parameters (Röthig et al., 2020). Especially in potentially compromised corals, the shift of bacterial communities is also an adaptive strategy that can improve holobiont physiology and play a critical functional role in maintaining nutrient cycling and supporting immunity (Osman et al., 2020). Therefore, the microbiome is generally thought to change its composition and function, thereby facilitating host adaptation and ecological plasticity in adapting corals to rapid environmental changes (Reshef et al., 2006; Torda et al., 2017).

However, multiple stressors associated with climate change, overfishing, and overexploitation may lower the resilience of corals to environmental changes in coastal ecosystems (Kennedy et al., 2015; Hughes et al., 2017). Recently, more than 25% of tropical coral reefs worldwide have been directly threatened by pollution from land-based sources (Martinez-Escobar and Mallela, 2019). In particular, the increasing flow of sewage into coastal ecosystems, driven by growing coastal populations, can lead to elevated nutrient concentrations that have detrimental effects on corals (Ziegler et al., 2016). The unprecedented rate of environmental change characterized by the Anthropocene has prompted pioneering studies exploring the possible role of the microbiome in coral holobiont plasticity (Biagi et al., 2020). It has been suggested that host-microbiome flexibility contributes to environmental robustness, while the underlying mechanisms remain unclear (Ziegler et al., 2019). Climate and anthropogenic stressors cause changes in the composition of coral bacterial communities, impairing the normal functioning of coral species and making them susceptible to opportunistic infections (Paulino et al., 2017). Despite the disturbance of pollution and development along the coast, some corals still can survive under conditions far from their natural habitat, which has invited scrutiny by scientists aiming to understand better how these corals respond to these conditions (Roitman et al., 2020). Therefore, it is essential to understand the effects of these stressors on the key members of holobionts. However, the extent to which microbial communities contribute to adapting coral hosts in a changing environment remains unknown (Hall et al., 2018). Coral microbial communities can be used as biomarkers of coral health and in response to environmental perturbations. Understanding how stressors alter the coral microbiome enables early management interventions in coastal coral ecosystems (Glasl et al., 2019; Roitman et al., 2020).

Coral reefs in the South China Sea (SCS) are distributed in areas ranging from nearshore coast to uninhabited, remote regions (Liao et al., 2021). Therefore, they provide a unique opportunity to investigate the relationship of coral holobionts to environmental disturbances. *Galaxea fascicularis* is widely distributed in the Indo-Pacific region, and it is an essential ecological and dominant species in the SCS (Zhou et al., 2017; Zhu et al., 2022a). Due to its resistance to environmental changes and stress, it can be used as an important model organism to study the response mechanism of coral holobionts to the environment. Furthermore, the development of high-throughput sequencing technologies has enhanced the understanding of the coral microbiome field. Previous studies have shown that temperature drives the local acclimatization of Symbiodiniaceae community structures *hosted* by *G. fascicularis* (Tong et al., 2017; Zhou et al., 2017). Meanwhile, the microbiome assemblages of *G. fascicularis* and other reef-building corals collected from different biogeographic regions of SCS are compared (Li et al., 2013; Cai et al., 2018). To better understand the potential impact of nearshore on the health and survival of corals and test whether microbial regulation supports the broad tolerance of disturbance in multiple stressor scenarios, we collected samples of *Galaxea fascicularis* from nearshore and offshore (less disturbed) sites to investigate microbial communities (Symbiodiniaceae and bacteria) and explore their relationship with environmental factors.

## Materials and methods

### Study area

**Figure 1:**
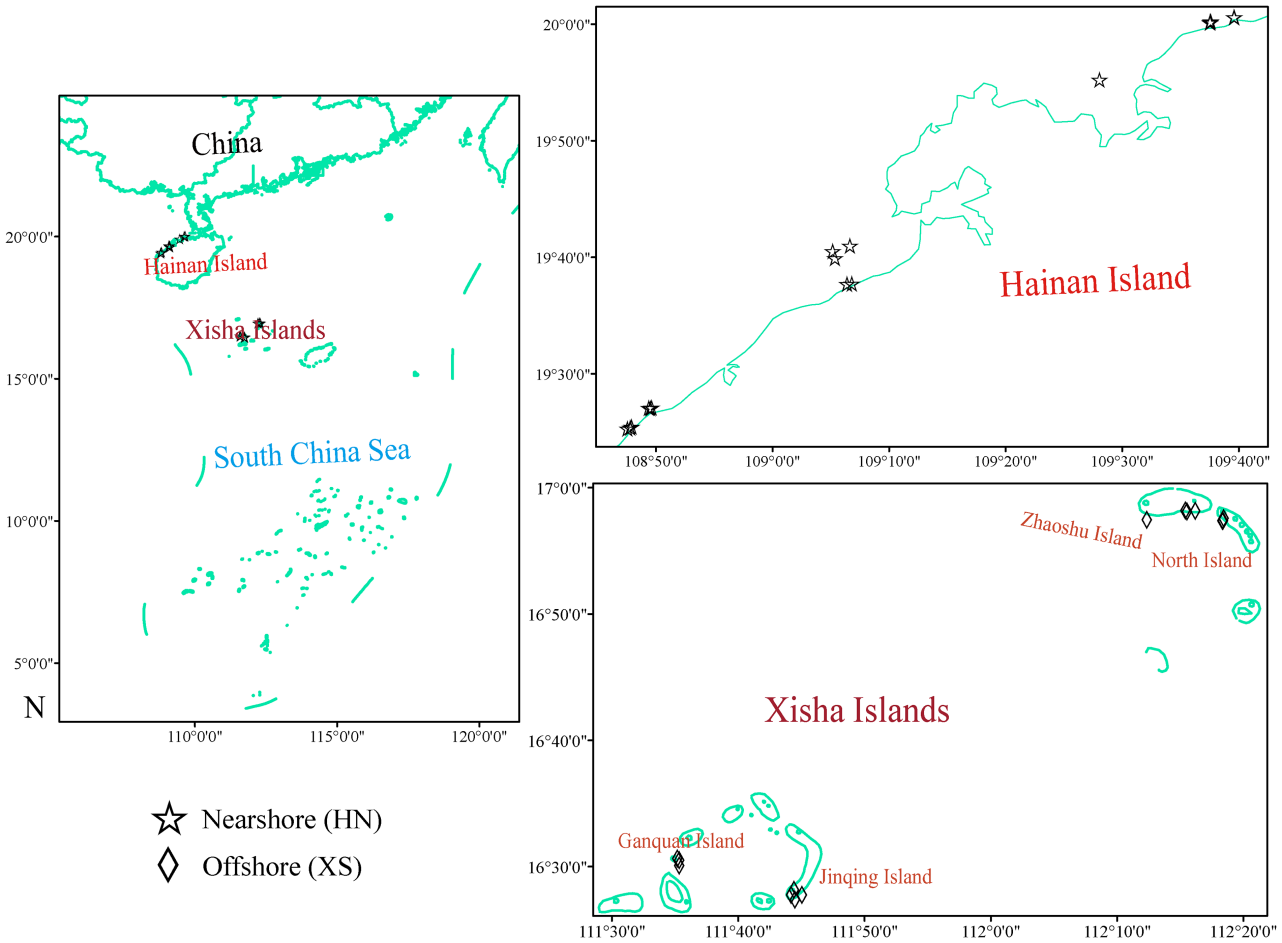
Study sites in Hainan Island (HN) and Xisha Islands (XS). HN represents typical nearshore coral reefs, and XS represents less-disturbed offshore coral reefs.

Hainan Island is located on the tropical northern edge of the Indo-Pacific Ocean in the SCS (18°09′–20°10′ N and 108°37′–111°03′ E; Figure 1), near the Coral Triangle, which is recognized as the center of coral reef biodiversity (Dong et al., 2017). Its environmental settings, including shallow water depths, tropical temperatures, and ample sunlight, are optimal for coral reef growth, and coral reefs are found on the island’s adjacent shores (Roder et al., 2013). However, Hainan Island has been overdeveloped since it became an international tourism island (Jiang et al., 2017). The process of coastal urbanization of Hainan Island has accelerated, sewage discharge, aquaculture effluents, and urban runoff have increased, and a large number of nutrients and organic matter have been discharged, leading to serious eutrophication and pollution to the coastal waters (Herbeck et al., 2013; Jiang et al., 2017). Since the 1980s, at least 50% of the marginal coral reefs on Hainan Island have been damaged by human activities, such as widespread coastal development and habitat destruction, and the live coral cover of the coastal reefs has declined severely (Hughes et al., 2013). Xisha Islands (15°40′N ~ 17°10′N, 110°E ~ 113°E) is located in the northwest of the SCS, about 300 to 400 kilometers away from Hainan Island. These tropical coral reefs of the Paracel Islands, including many atolls and/or island reefs in the central SCS, are very suitable for coral growth and development (Qin et al., 2019). In contrast, as far-sea islands far away from their surrounding densely populated continents, Xisha coral reefs are relatively less affected by human activities and have well-developed oligotrophic tropical marine coral reefs and relatively high species diversity (Qin et al., 2019).

### Sample collection and environmental parameters

From July to August 2021, the scleractinian coral *G. fascicularis* remaining visually healthy was collected on the northwest coast of Hainan Island and Xisha Islands in the SCS (Figure 1), and samples of 18 and 14 colonies were collected, respectively (Supplementary Table 1; Supplementary Figure 1). Coral fragments were obtained from a depth range of 2–10 m by self-contained underwater breathing apparatus diving (SCUBA) with hammers and chisels in sterile bags, and all fragments were stored at −20°C and transported to the laboratory. Each sample was subsampled and divided into two parts, one for physiological measurements and the other for DNA extraction. General environmental parameters, including water temperature, salinity, dissolved oxygen, pH, and turbidity, were determined according to the standard methods described in our previous study (Zhu et al., 2022a). Seawater samples were collected near coral samples, filtered immediately (Whatman GF/F), and stored at low temperatures. In the laboratory, dissolved nutrients (nitrite, nitrate, ammonia, and phosphate) were determined using an automatic nutrient analyzer, as previously described (Zhu et al., 2022b).

### Physiological analysis

The coral surface was rinsed with a Water-Pik containing seawater (filtered through a 0.45-μm filter) until only the white coral skeleton remained. The volume of the rinsed solution was measured in a measuring cylinder, then 10 ml of rinsed solution was measured, and the number of zooxanthellae was counted and recorded under a microscope using a blood cell counter (eight repetitions; Fitt et al., 2000). For the analysis of total biomass, the tissue homogenate was dried at 60°C for at least 24 h and then weighed, and the dry weight was recorded after 12 h of constant-temperature drying (Fitt et al., 2000). Subsequently, the specimens were burned in a muffle furnace at 500°C for at least 4 h and weighed. The ash weight was recorded after 12 h of constant-temperature drying, and the biomass content of coral was the difference between weights before and after weighing. The coral surface was wrapped with aluminum foil and weighed, and the unit surface area of the coral was determined according to the correlation between the aluminum foil weight and the surface area (Zhu et al., 2022c). The final results were expressed as zooxanthellae density (cells/cm^2^) and biomass (mg/cm^2^) per unit of coral surface area.

### Sequencing of symbiodiniaceae and bacteria

Total DNA from 32 coral samples was extracted using the Mobio Powerwater DNA extraction kit according to the manufacturer’s instructions. The specific primers itsintfor2 (5′-GAATTGCAGAACTCCGTG-3′) and ITS2 reverse (5′-GGGATCCATATGCTAAGTTCAGCGGGT-3′) were used to for amplifying the ITS variable region of Symbiodiniaceae rRNA gene (Grottoli et al., 2004). The bacterial full-length 16S rRNA gene was amplified using primers 27 f (5′-AGGRGTTYGATYMTGGCTCAG-3′) and 1492 R (5′-RGYTACCTTGTTACGACTT-3′; Pootakham et al., 2021). PCR amplification was performed using GeneAmp® 9700 thermal cycle controller (Thermo Fisher Scientific, United States) under the following conditions: 95°C for 30 s, followed by 35 cycles of 95°C for 10 s, 60°C for 30 s, 72°C for 45 s, and a final extension at 72°C for 10 min. According to the standard protocol of Majorbio Biopharm Technology Co., Ltd. (Shanghai, China), the GeneJET Gel Extraction Kit (Thermo Scientific, United States) was used to purify PCR products. The Symbiodiniaceae were sequenced using a 2 × 300 bp model on the Illumina Miseq platform, and bacteria were sequenced using the PacBio Sequel single-molecule real-time sequencing system.

The high-quality reads were clustered into operational taxonomic units (OTUs) at 97% identification. BLASTn was used to select the most abundant OTU sequences as representative sequences for comparison with the ITS2 database (Shi et al., 2021), and non-Symbiodiniaceae OTUs were removed. Bacterial taxonomy was assigned by comparison with the Silva 16S rRNA gene database (V138) using the Ribosomal Database Project (RDP) classifier. Finally, OTU data were resampled to the lowest sequence of Symbiodiniaceae and bacterial community for downstream analysis.

### Data analysis

All data analyses were performed in R version 4.0.3.^1^ Spatial patterns in environmental parameters were analyzed by principal component analysis (PCA) and Student’s *t*-test to determine the spatial differences between the two regions. The Shannon index of Symbiodiniaceae and bacteria was calculated in the “vegan” package. After testing the data normality and homoscedasticity of variances, the difference in diversity index between the two groups was compared by t-test (*p* < 0.05). Non-metric multidimensional scaling (NMDS) and analysis of similarity (ANOSIM) based on the Bray–Curtis dissimilarity index were performed to investigate the patterns of microbial community structure. Community similarity (1- dissimilarity of the Bray–Curtis distance) and log-transformed data of geographic distance were used to study the distance attenuation relationship (DDR). Based on the results of the detrended correspondence analysis (DCA), Redundancy analysis (RDA) was used to determine the association between the communities and environmental factors (Lai et al., 2022). LEfSe analysis was used to determine the biomarkers with different abundances from the phylum to genus levels among groups. Based on the microbial classification information, the potential bacterial functions of different groups were predicted by the Functional Annotation of Prokaryotic Taxa program (FAPROTAX) using the default settings (Louca et al., 2016).

Finally, we constructed a species co-occurrence network based on Spearman’s correlation matrices. To reduce the rare bacterial OTUs in the dataset, only the OTUs appearing in at least half of the samples were retained (Barberán et al., 2012). All paired Spearman correlations of OTUs were calculated using the “Hmisc” package. Robust correlations were considered valid when Spearman’s correlation coefficient (r) > |0.7| and statistically significant (*p* < 0.01). Finally, the “igraph” package and Gephi (v.0.9.2; https://gephi.org/) were used to build and visualize co-occurrence networks (Csardi and Nepusz, 2005). The topological characteristics of the network were described by nodes, links, average path distance, average clustering coefficient, and modularity (Zhu et al., 2022b). Sub-networks were generated for each sample from the meta-community network by preserving the OTUs presented in each site using the subgraph function in the “igraph” package (Ma et al., 2016). Each sub-network was grouped by sampling location, and the Wilcoxon rank-sum test was used to identify different network-level topological features between sampled regions. The nodes with a high degree were identified as the keystone taxa in the co-occurrence network (Banerjee et al., 2018). The linear regression analysis was used to establish the relationship between environmental parameters and bacterial diversity, co-occurrence topological features, and relative abundance of keystone taxa.

## Results

### Environmental differences and coral physiology

**Figure 2:**
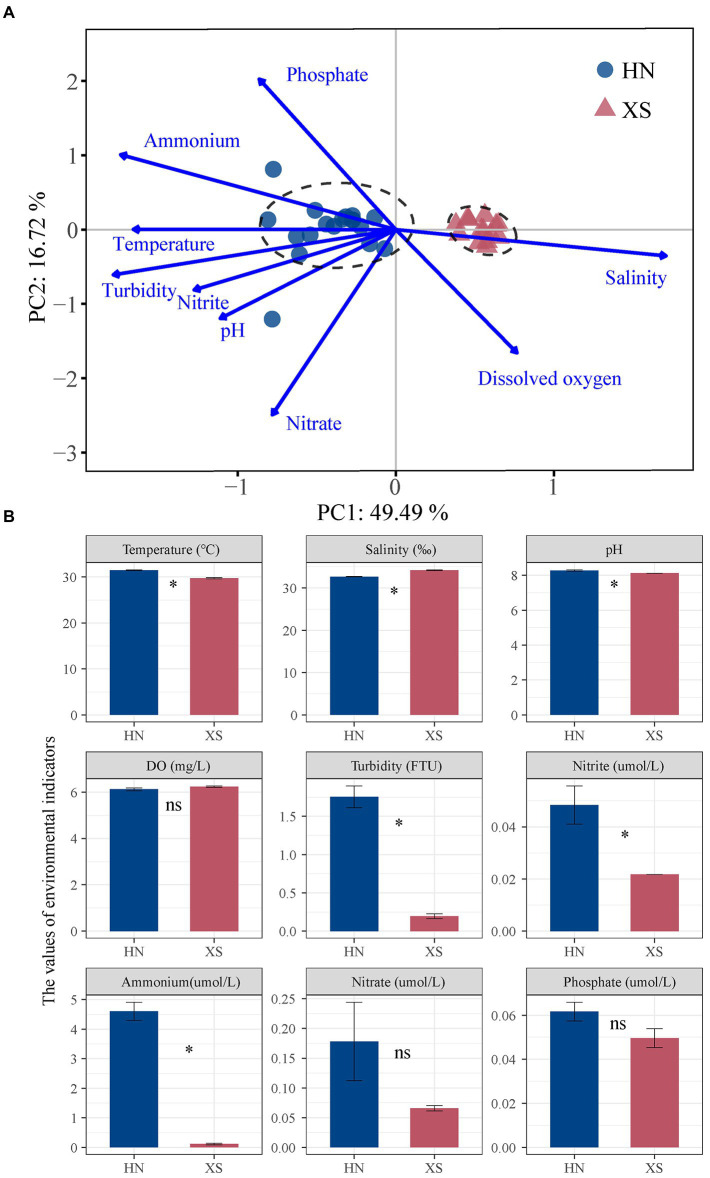
Analysis of the environmental parameters in the sampling sites. PCA results for environmental variables **(A)** in Hainan Island (HN) and Xisha Islands (XS). The physicochemical parameters *in situ* **(B)**. Asterisks indicate significant differences in the same environmental parameter between HN and XS.

The PCA results and permutational multivariate analysis of variance (PERMANOVA) showed that the environmental conditions of the northwest coast of Hainan Island and the Xisha Islands were best differentiated (Figure 2A; *p* = 0.001). Among them, the mean temperature of seawater at the nearshore station (HN) was 31.47 ± 0.12°C, while the mean temperature at the offshore station (XS) was 29.73 ± 0.15°C (Figure 2B). The salinity *in situ* of HN (32.66 ± 0.06‰) was significantly lower compared with XS (34.21 ± 0.10%; *p* < 0.001). The mean values of pH, turbidity, ammonium, and nitrite at HN were significantly higher compared with XS (8.30 vs. 8.12; 1.75 NTU vs. 0.20 NTU; 4.60 umol/L vs. 0.11 μmol/L; 0.05 μmol/L vs. 0.02 μmol/L; *p* < 0.001). For dissolved oxygen, nitrate, and phosphate contents, there was no significant difference between the two sea areas. In general, compared with the offshore coral reef sites in the Xisha Islands, the temperature and water quality of the offshore coral reefs in northwest Hainan were higher. It was worth noting that the symbiont density (2.27 ± 0.23 ·10^6^ cells/cm^2^) in the healthy state collected at the HN station was slightly higher compared with the XS station (1.82 ± 0.13 ·10^6^ cells/cm^2^; Supplementary Figure 2). The tissue biomass at HN (6.89 ± 0.54 mg/cm^2^) was slightly lower compared with XS (8.11 ± 0.76 mg/cm^2^). This was interesting because there was no significant difference between the two in different sea areas.

### Symbiodiniaceae community composition and driving factors

**Figure 3:**
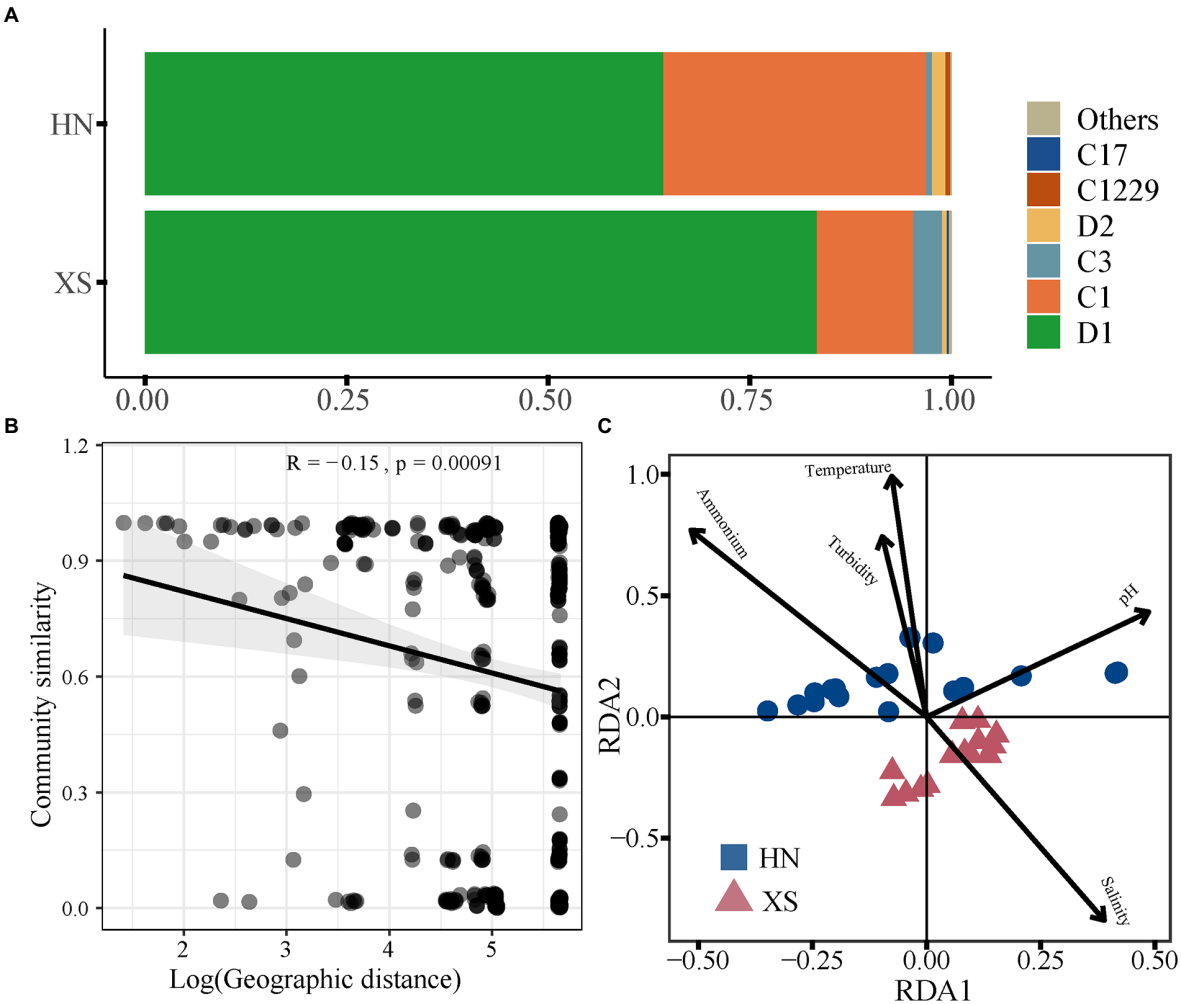
Symbiodiniaceae community composition and driving factors. Heatmap of Symbiodiniaceae community **(A)**. Relationships between the Bray–Curtis similarities of Symbiodiniaceae community and geographic distance **(B)**. RDA ordination shows associations between the Symbiodiniaceae community and environmental factors **(C)**.

The symbiotic Symbiodiniaceae genotypes were analyzed by Illumina high-throughput sequencing in Hainan Island and Xisha Islands (HN: n = 18; XS: n = 14). After quality control, 465,889 sequences belonging to the Symbiodiniaceae family were identified, and these high-quality sequences were clustered to form 25 Symbiodiniaceae OTUs. The Shannon index of Symbiodiniaceae was lower in HN (0.36 ± 0.04) compared with XS (0.55 ± 0.15), while there was no significant difference (Supplementary Figure 3A). NMDS analysis indicated strong individual heterogeneity in Symbiodiniaceae community composition but less separation between HN and XS (ANOSIM: *p* > 0.05; Supplementary Figure 3B). The Symbiodiniaceae types of all coral samples were dominated by ITS2 sequences from Durusdinium and Cladocopium. Among them, D1 was the most prevalent type in the composition of the Symbiodiniaceae of HN (62.27%) and XS (83.31%), followed by the relative abundance of the second dominant species C1, which was 32.56 and 11.96%, respectively (Figure 3A). There was a significant negative linear correlation between Symbiodiniaceae community similarity and geographic distance (Figure 3B; R = −0.15, *p* < 0.001). Using the RDA of Symbiodiniaceae community composition and the screened important influencing factors (*F* = 3.735, *p* = 0.007), the two axis pairs could explain 41.79% of the overall variation in the Symbiodiniaceae composition (Figure 3C). The results showed that pH (14.2%), ammonium (10.15%), temperature (7.75%), salinity (5.71%), and turbidity (3.98%) significantly affected the Symbiodiniaceae community.

### Bacterial community composition and changes in bacterial functional groups

**Figure 4:**
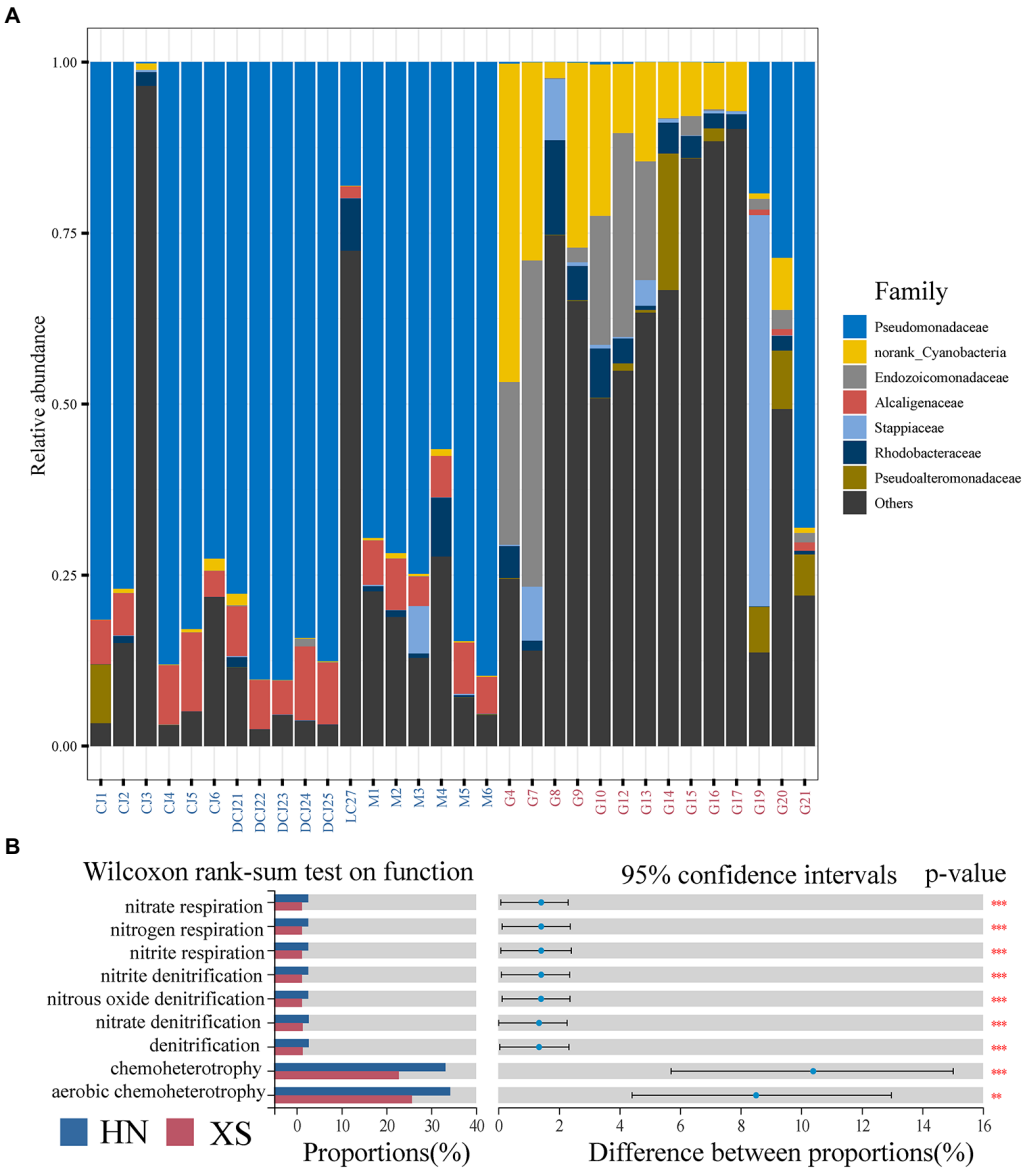
Composition of bacterial community changes in bacterial functional groups.Taxonomic composition at the prokaryotic family level **(A)**. The differences in functional groups were analyzed using Wilcoxon rank-sum test **(B)**, and *, **, and *** denote value of p less than 0.05, 0.01, and 0.001, respectively.

At the family level (Figure 4A), the bacterial community of HN corals was dominated by Pseudomonadaceae (>50%), followed by Alcaligenaceae and Rhodobacteraceae (abundance >1%). Interestingly, norank Cyanobacteria, Endozoicomonadaceae, and Rhodobacteraceae were the dominant groups in the XS samples, and the relative abundance was found to be significantly higher compared with the HN samples. In addition, Pseudomonadaceae, Stappiaceae, and Pseudoalteromonadaceae were also dominant bacteria with a relative abundance higher than 1%. We further used LEfSe analysis to identify environmental indicator taxa in different regions (Supplementary Figure 4). The LDA scores indicated that HN coral samples were rich in Pseudomonas, Alcaligenaceae, Burkholderiales, and Coxiellaceae. Cyanobacteria, Endozoicomonadaceae, Rhizobiales, Alteromonadales, Cytophagales, Vibrionales, Acidimicrobiia, Actinobacteriota, Vibrio, Pseudoalteromonadaceae, Burkholderiaceae, Ralstonia, Flavobacteriales and Rhodobacteraceae were enriched in XS coral samples.

Based on FAPROTAX functional database, bacterial functions of different stations were identified. PCA results showed significant differences in microbial functional characteristics between HN and XS samples (Supplementary Figure 5A). In general, aerobic chemoheterotrophy and chemoheterotrophy were the main functional groups in all samples, with a relative abundance of more than 20%, especially higher in HN samples than XS (Figure 4A). In addition, the main functions with a relative abundance higher than 1% included denitrification, nitrate respiration, nitrogen respiration, nitrite respiration, nitrite denitrification, nitrous oxide denitrification, and nitrate denitrification, and their abundance in HN was significantly higher compared with XS. Finally, we found that these functional groups were significantly positively correlated with temperature, turbidity, and ammonium concentration but significantly negatively correlated with salinity (Supplementary Figure 5B).

### Bacterial community associated with environmental factors

**Figure 5:**
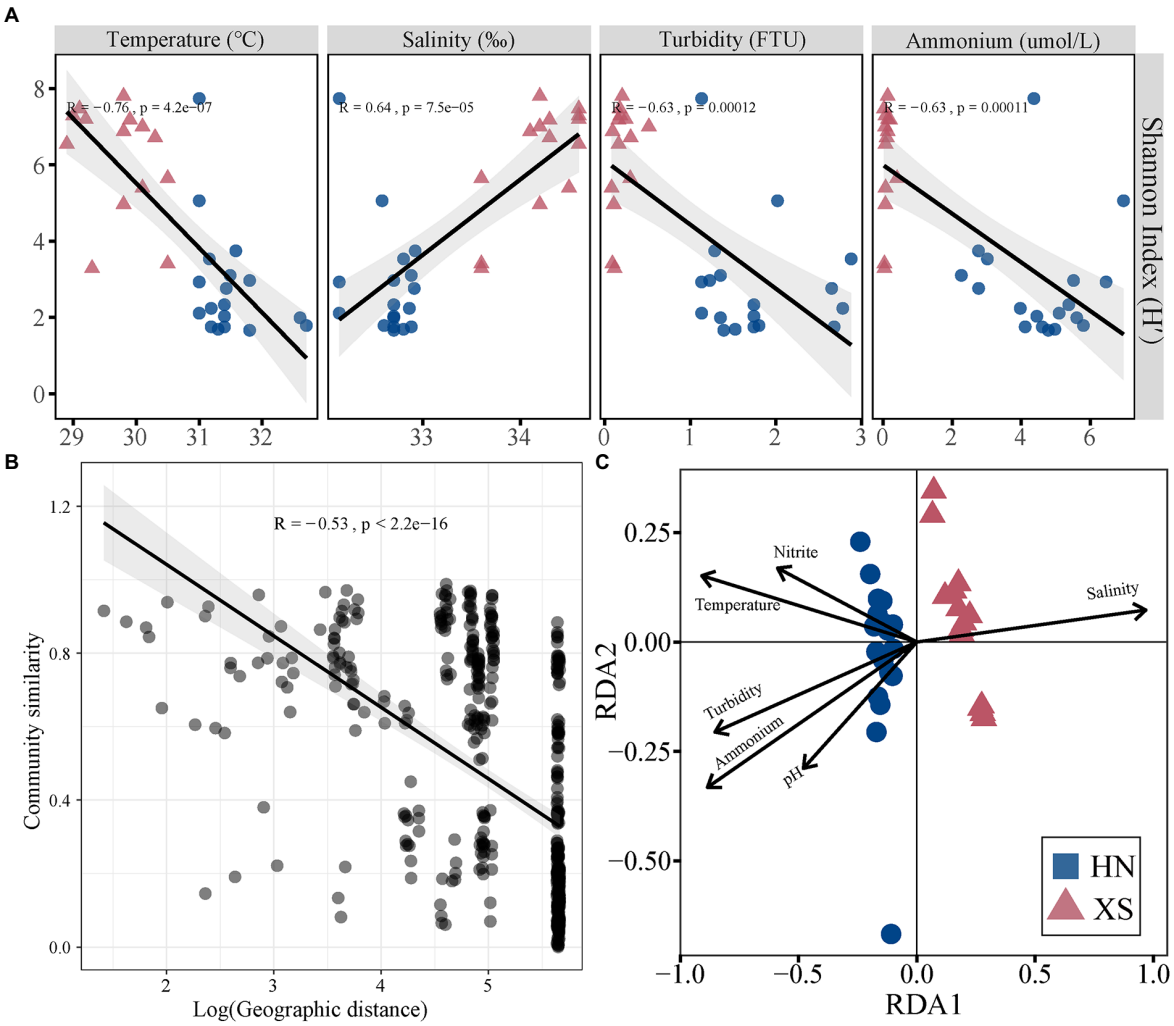
Correlation between different factors and bacterial community structure. Relationships between Shannon index of the bacterial community and environmental factors **(A)**. Relationships between the Bray–Curtis similarities of bacterial community and geographic distance **(B)**. RDA ordination shows associations between bacterial community and environmental factors **(C)**.

The Shannon diversity index of coral-associated bacteria was significantly lower at HN compared with XS (*t* = 6.35, *p* < 0.001; Supplementary Figure 6A). NMDS analysis revealed a significant difference in bacterial communities between HN and XS samples (PERMANOVA: R^2^ = 0.35, *p* < 0.001; Supplementary Figure 6B), and the distances between samples within the HN group were more dispersed (ANOSIM: R = 0.743, *p* = 0.001). The bacterial Shannon diversity index was significantly decreased with increasing temperature, turbidity, and ammonium content but increased with increasing salinity (Figure 5A). A distance decay pattern of similarity was found in bacterial communities, where the composition similarity of the communities was significantly decreased with increasing geographic distance (R = −0.53, *p* < 0.001; Figure 5B). The overall results of RDA were significant for the coral bacterial community (*F* = 3.23, *p* = 0.001). The RDA results showed that explanatory variables accounted for 58.2% of the total variation, among which salinity (11.66%), temperature (9.15%), ammonium (8.69%), turbidity (7.25%), nitrite (3.68%), and pH (3.24%) could significantly affect the coral bacterial community (Figure 5C).

### Bacterial co-occurrence network and influencing factors

**Figure 6:**
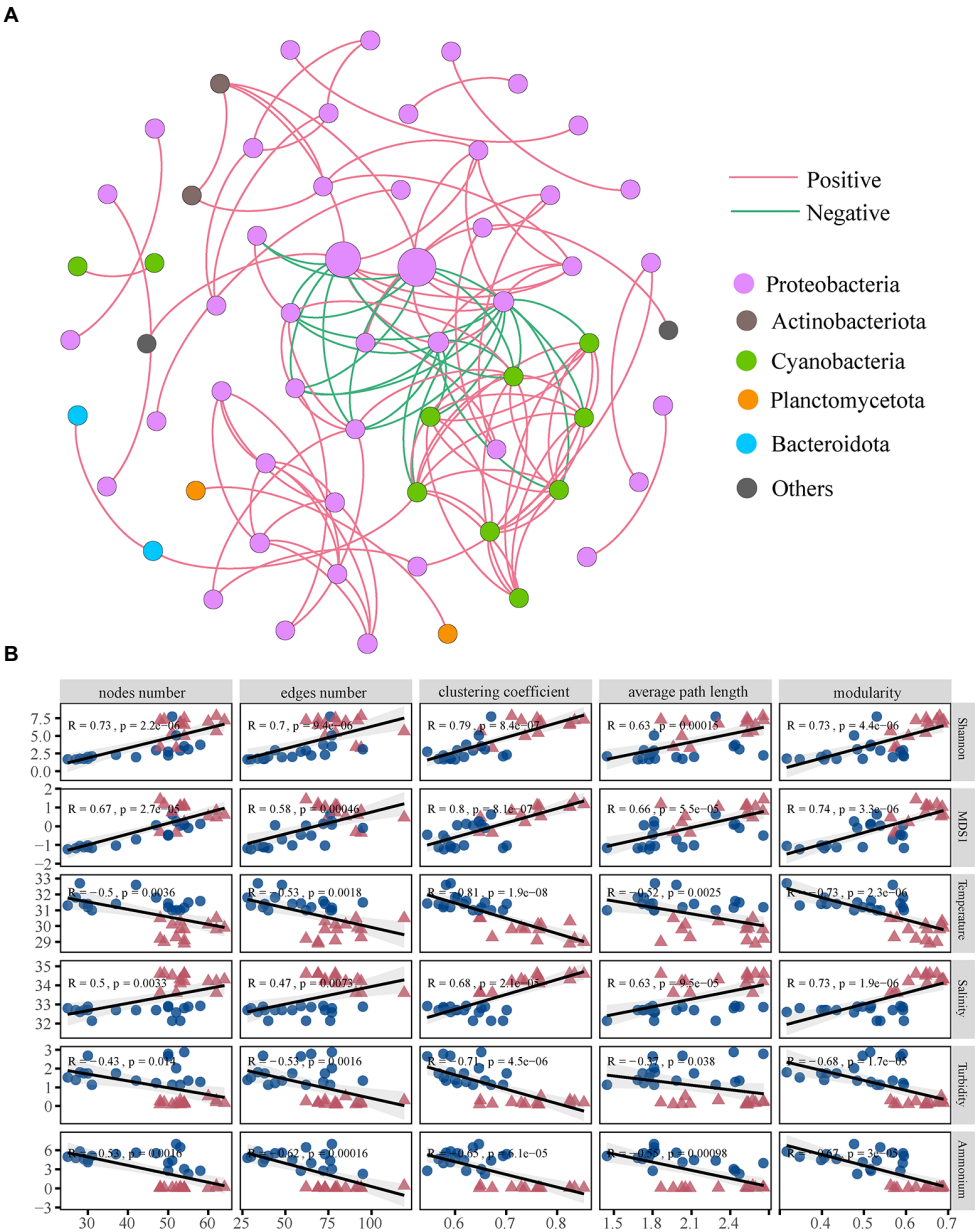
The co-occurrence network interactions and topological features. Co-occurrence networks for bacterial communities **(A)**. Each node represents a species (OTU), and each edge connecting two nodes indicates the relationship between these two species. Different node colors represent OTUs from different phyla. Node size is proportional to node degree. Red edges represent positive correlations, and blue edges represent negative correlations. Significant correlation between environmental parameters and network-level topological features **(B)**.

Based on strong and significant correlations, we constructed a network pattern of coral bacterial co-occurrence (Figure 6A). A total of 62 nodes and 124 edges were found. The average path length was 2.554 edges, the clustering coefficient was 0.647, and the modularity was 0.590 (Supplementary Figure 7A). We further examined the bacterial sub-network topological features and influencing factors. The results of the Wilcoxon rank-sum test showed that the scale (number of nodes and edges), connectivity (average path length and clustering coefficient), and modularity of the XS bacterial network were higher compared with HN (*p* < 0.05). Moreover, these parameters were significantly positively correlated with bacterial diversity (alpha and beta diversity) and salinity but significantly negatively correlated with temperature, turbidity, and ammonium (Figure 6B). The “hubs” that often appear in network analysis usually refer to the species with a high mean degree in the community. We could identify the members of Pseudomonadaceae, Alcaligenaceae, Rhizobiaceae, and Cyanobacteria as keystone taxa. Further analysis found that these taxa were significantly correlated with environmental factors (Supplementary Figure 7B).

## Discussion

### Physiology tolerance of inshore coral and specificity of dinoflagellate communities

Previous studies have shown that zooxanthellae density and tissue biomass are good indicators of coral health status and environmental stress tolerance (Fitt et al., 2000; Wooldridge, 2014). Through the *in-situ* measurement of environmental data, we found that the water temperature, nutrients, and turbidity of offshore coral reefs in Hainan Island were higher compared with Xisha Islands (Figure 2). However, there were no significant differences in coral zooxanthellae density and tissue biomass between different sea areas (Supplementary Figure 2), which might be explained by the following aspects. During the study period, offshore coral reefs had low turbidity and oligotrophic conditions, which might limit the reproduction of symbionts, and the high light intensity of solar radiation might lead to a decreased zooxanthellae density (Qin et al., 2019). In contrast, offshore corals in Hainan Island were at moderate nutrient concentrations (DIN: < 10 μmol L^−1^, DIP: < 0.35 μmol L^−1^), and the abundant inorganic nitrogen content stimulated the symbiotic algae to utilize nutrients for their growth and reproduction (Wiedenmann et al., 2013). Therefore, despite elevated temperatures on the nearshore, moderate nutrient concentrations can provide physiological benefits to coral health and mitigate the adverse effects of these stressors (Dobson et al., 2021; Zhu et al., 2021). The environments of inshore and offshore corals differed markedly, while the physiology of the corals suggested that they had broader resistance and physiological plasticity.

The flexibility of host-endosymbiont associations is a strategy for corals to survive across biogeographical regions and specific local environmental stresses (Osman et al., 2020). Durusdinium (D1) and Cladocopium (C) were the primary symbiotic algae in this study (Figure 3A), which was consistent with previous studies (Zhu et al., 2021). In general, Durusdinium is assumed to withstand stress because it can exist in coral reef habitats with high environmental disturbances (e.g., fluctuations in temperature, salinity, nutrients, or sediments; Chen et al., 2005; Kennedy et al., 2015). In particular, prolonged exposure to tropical environments forces corals to associate with the prominent thermotolerant clade D1, making them more resistant to heat stress (Hume et al., 2016). Furthermore, clade C1 is also the most prevalent symbiont in many Symbiontaceae communities, associated with corals that survive in high temperatures and intense light (Ng and Ang, 2016; Moghaddam et al., 2018). C1 usually provides the host with a higher amount of carbon and growth rate, which increases the physiological resistance and plasticity to enhance the fitness of the host (Cantin et al., 2009). In addition, low symbiotic diversity in Hainan Island is also a cost-effective adaptation strategy to cope with highly fluctuating suboptimal environmental conditions in disturbed inshore coral reefs (Ng and Ang, 2016).

Previous studies have shown that stressful environments can exert strong selective pressure on corals, which select only specific well-adapted symbiotic lineages, reducing their symbionts (Saad et al., 2022). We found a significant negative linear relationship between Symbiodiniaceae community similarity and geographic distance (Figure 3B), which might be related to their dependence on ocean currents for dispersal and limited by geographic distance (Wiedenmann et al., 2013). Environmental conditions are known to shape the distribution patterns of symbionts at larger spatial scales (Ziegler et al., 2017a), with the temperature being the primary factor driving the coral-symbiont association in the tropical SCS (Tong et al., 2017). Other environmental factors (especially nutrients and turbidity) can also be the main factors affecting the spatial distribution of Symbiodiniaceae (Figure 3C), suggesting that human activities also affect the symbiotic association (Gong et al., 2018). In particular, *G. fascicularis* appears to be parasitizing more adaptive symbiotic algal populations in tropical coral reefs, both inshore and offshore, which may help them dominate future climate change.

### High plasticity of habitat-associated bacteria

Corals have highly diverse bacterial distributions in each habitat, and we found that bacterial indicator species containing high relative abundances were significantly associated with habitat. The dominant bacteria in HN coral samples were Pseudomonadaceae, Alcaligenaceae, and Rhodobacteraceae, while norank Cyanobacteria, Endozoicomonadaceae, Rhodobacteraceae, Pseudomonadaceae, Stappiaceae, and Pseudoalteromonadaceae were the dominant bacteria in XS coral samples (Figure 4). These dominant taxa are all coral symbionts that have been reported in previous studies and involved in the production of antipathogenic compounds in trophic pathways (i.e., nitrogen fixation, organic carbon degradation, and the cycling of Dimethylsulfoniopropionate and dimethylsulfide; Lesser et al., 2004; Raina et al., 2010; Lema et al., 2014; Neave et al., 2017; Bernasconi et al., 2019; Maire et al., 2021). LDA scores suggested that inshore corals were enriched in taxa, such as Pseudomonas of Pseudomonadaceae and Alcaligenaceae of Burkholderiale (Supplementary Figure 4), and the high abundance of these bacteria might indicate thermal anomalies and trophic environments in the waters surrounding Hainan Island. Among them, Pseudomonas is an N-cycling bacterium associated with the denitrification process and is involved in DMSP/DMS metabolism, possibly enabling corals to cope with increased oxidative stress in symbiotic algae (Raina et al., 2010). In addition, the dominant Pseudomonas on nearshore is considered to be the indicator microorganism for algae-dominated coral reefs (Kelly et al., 2014). Burkholderia is ubiquitous in the microbial niches of various coral species and is involved in various nitrogen metabolism processes (Ainsworth Tracy et al., 2015; Berkelmann et al., 2020). Burkholderia is also known for its potential to degrade phenolic and aromatic compounds in the environment (Pérez-Pantoja et al., 2012), which may be related to anthropogenic pollution in coastal coral reefs in Hainan. In addition, these bacteria may adapt to ammonium concentrations through nitrification and denitrification processes, providing some beneficial functions in connection with tolerance patterns in coastal corals (Peixoto et al., 2017).

Our indicator species supported the concept of local domestication (Osman et al., 2020), and the offshore sites contained more significantly related bacterial taxa, including Cyanobacteria, Rhizobiaceae, Vibrio, Ralstonia, and Stappiaceae. These bacteria are known to fix nitrogen, produce ammonia, and participate in the carbon and ammonia cycle, which may provide the coral with essential functions, such as fixing nutrients in the nutrient-poor Xisha coral reef waters (Chimetto et al., 2008; Lema et al., 2014; Ainsworth Tracy et al., 2015; Bernasconi et al., 2019). Endozoicomonadaceae, the other dominant group in Xisha Islands, are presumed beneficial bacteria related to global coral health (Dunphy et al., 2019). They have potential functions in the carbohydrate cycle, nitrogen cycle, heat stress protection, and antibacterial activity, which can help corals adapt to the environment of high radiation and low nutrition in the Xisha Islands (Damjanovic et al., 2020). Rhodobacteraceae is generally considered a member of the coral microbiome, which is usually related to the nitrogen, carbon, and sulfur cycles in corals (Lesser et al., 2018; Pootakham et al., 2021). They can also produce and significantly regulate reactive oxidative species (ROS) in their surroundings, which may play a key role in improving the resistance of the whole organism to heat stress (Osman et al., 2020). Interestingly, although Vibrio, Rhizobacteriaceae, and Rhodobacteriaceae are symbiotic and opportunistic coral pathogens, they are also thought to play a role in the nutrient cycling of the holobionts under normal conditions (Ainsworth Tracy et al., 2015; Pootakham et al., 2021). In summary, those bacteria involved in nitrogen fixation and recycling play a potential role in host nutrient metabolism and are critical in maintaining symbiont stability in an oligotrophic environment.

### Effects of environment on bacterial community and ecological function

Coral-associated bacterial alpha diversity may be increased due to regional and global stressors, and stressful events appear to disrupt microbiome function (McDevitt-Irwin et al., 2017). Similarly, the bacterial alpha diversity was significantly decreased with increasing temperature, turbidity, and ammonium content in this study (Figure 5). Loss of beneficial bacterial diversity possibly affected the resilience of coral holobiont to buffer the impacts of environmental perturbations since reduced bacterial diversity is known to diminish the ability of corals to resist infection, assimilate nutrients, and maintain the aggregate function (Pollock et al., 2019). At the same time, increased dispersion of bacterial communities was found among corals in the nearshore environment compared with the more stable communities in offshore corals, which was consistent with the Anna Karenina principle that stressors lead to unstable community states (Zaneveld et al., 2017). Coral bacterial communities change with fluctuations in environmental conditions (e.g., temperature, salinity, and nutrient availability; McDevitt-Irwin et al., 2017). Consistent with our results, temperature and salinity are important factors in determining bacterial community in a variety of previous reports (Röthig et al., 2016; Ziegler et al., 2017b). The coral reefs located in nearshore regions are affected by human activities. Thus, surrounding eutrophication was also the main environmental driver of coral-associated bacterial community structure (Staley et al., 2017; Ziegler et al., 2019). In addition to reef habitat-related environmental factors, we also found significant distance-decay relationships in bacterial communities (Figure 5). For example, the characteristics of coral-related bacterial communities in an urbanized marine environment show great differences in a small geographical range, and the water flow and wind direction affect the observed differences in the bacterial community (Wainwright et al., 2019). These results suggest that regional processes, such as dispersal limitation and environmental heterogeneity, play an essential role in structuring coral microbial communities (Dunphy et al., 2019).

Functional prediction analysis showed that the carbon cycle and nitrogen cycle were the main functional groups in all samples (Figure 4), which was consistent with the above-mentioned results of potential function analysis of dominant and indicator species. These results further suggested that although environmental stress reduced bacterial diversity and changed community structure, the critical functions of symbionts always existed. The flexibility of the microbiome is considered to be related to the fact that high community diversity and/or functional redundancy within the bacterial community can promote the retention of essential functions under pressure even if the composition of the bacterial community changes (Voolstra and Ziegler, 2020). Interestingly, the abundance of these functions in nearshore samples was significantly higher, and the changes in specific bacterial population functional groups were particularly related to the environmental impacts, such as nutrients, temperature, and turbidity (Supplementary Figure 5). It is expected that local environmental conditions will select specific metabolic pathways in microorganisms that play an essential role in coral health, and the metabolic potential of microbial communities is most closely related to location (Kelly et al., 2014). Supported by measurable coral physiological indicators and predictive functions, the microbiome reorganization caused by environmental changes in this study might support the probiotic hypothesis (Reshef et al., 2006). *G. fascicularis* exhibits a high degree of plasticity and flexibility in bacterial communities under environmental changes, which can counteract possible negative dysbiosis and influence coral health (Ziegler et al., 2019).

### Nearshore environment reduces the complexity of bacterial network and changes the abundance of keystone species

Microbial networks provide insights into complex biological interactions and their response to environmental factors (Barberán et al., 2012; Morriën et al., 2017). Our study emphasized how environmental gradients affected the network structure of coral-associated bacteria, and the network became simpler with the increase in temperature, turbidity, and nutrients (Figure 6). It has been reported that high temperature changes the mode of microbial interaction (Zhu et al., 2022c), and the coral reef network that is more vulnerable to organic pollution lacks complexity (Leite et al., 2018), which is consistent with the findings in this study. The availability of nutrients in offshore seawater was decreased, and the coral bacterial network in the oligotrophic environment became more complex, which might enhance the stronger cooperation and nutritional interaction between coral microbial functional groups (Röttjers and Faust, 2018). Network connectivity plays a vital role in microbiome stability and ecosystem versatility, and higher connectivity is considered a rapid response to environmental changes (Morriën et al., 2017). The simpler network structure and smaller connectivity in nearshore coral reduced the efficiency of resource and information transfer, which was detrimental to the coral host’s nutrient use efficiency and ability to cope with environmental fluctuations.

Meanwhile, the complexity and connectivity of the network are generally positively correlated with the stability of the community (Mougi and Kondoh, 2012), which is further supported by the evidence in this study that the network module indicator was lower in a nearshore environment. In addition, the modularization of the microbial co-occurrence network not only reflects the stability of microbial communities but also determines the complementarity and redundancy of microbial groups (Allison and Martiny, 2008; Hernandez et al., 2021). In this sense, the higher complexity of the offshore network might indicate that the coral microbiome was more resistant to environmental stresses. We observed a positive correlation between bacterial α and β diversity and microbial network complexity and connectivity (Figure 6). Environmental perturbations might reduce the bacterial diversity of inshore corals, thus affecting network complexity and stability. This finding suggested that microbial interactions could contribute to microbial community diversification by acting as a selective force. Therefore, intrinsic factors, such as microbe-microbe interactions, are also potential major drivers of coral microbial community structure and holobiont homeostasis.

Key microbial taxa are highly related taxonomic groups that may confer greater biological connectivity to communities and can serve as indicators of community shifts (Herren and McMahon, 2018; Wagg et al., 2019). Our results showed that keystone taxa had critical ecological functions in the microbial community (Supplementary Figure 7). Pseudomonadaceae have different abilities important to corals (Leite et al., 2017). Various members of the order Rhizobacteria and Burkholderia have been identified as key taxa in diverse ecosystems, and these bacteria are known to play critical roles in nitrification and nitrogen fixation (Banerjee et al., 2018). Photosynthetic bacteria (cyanobacteria) can facilitate the survival of corals in oligotrophic environments by supplying nitrogen and carbon to coral reefs (Lesser et al., 2004). In our present study, network topological features and key taxa were significantly associated with environmental factors (Supplementary Figure 7). The key taxa identified here served as indicators of coral responses to environmental changes, given their marked reactions to the environment and their essential roles in microbial structure and function (Banerjee et al., 2019). Overall, coral bacteria closely related to carbon, nitrogen, and sulfur cycles were identified as keystone species, suggesting that the environment altered microbially mediated biogeochemical cycles, thereby affecting the physiological state of corals. Therefore, it could be inferred that shifts in coral symbiotic bacterial interactions and keystone species were also essential for coral hosts to cope with nearshore stress environments.

## Conclusion

As climate change and local pollution continue to devastate marine environments, it is vital to understand the local acclimatization of coral holobionts in response to multiple stressors. In the present study, we revealed the environmental flexibility and ecological adaptation of *Galaxea fascicularis* from a microbial perspective. First, complex nearshore environment suggested high physiological plasticity to support coral tolerance to environmental stress. Second, coral had low Symbiodiniaceae diversity and generalist symbionts to cope with the local suboptimal-environmental conditions. Third, flexibility in the coral-associated bacteria appeared to contribute to holobiont function and underpin the robustness of this broadly distributed coral. Finally, bacterial networks exhibited lower complexity and associativity in stressful environments but induced changes in keystone taxa supporting carbon and nitrogen cycling. Therefore, our results provided new insights into the response of coral holobiont to various stressors and highlighted the need to evaluate their consequences for coral acclimatization.

## Data availability statement

The datasets presented in this study can be found in online repositories. The names of the repository/repositories and accession number(s) can be found at: https://www.ncbi.nlm.nih.gov/, PRJNA 810356, PRJNA855370, PRJNA855369, and PRJNA 810598.

## Author contributions

WZ: conceptualization, investigation, methodology, and writing-original draft. MZ, XL, JX, HW, and RC: investigation. XL: conceptualization, writing-review and editing, and supervision. All authors contributed to the article and approved the submitted version.

## Funding

This work was financially supported by the National Natural Science Foundation of China (42076108, 42161144006 or 3511/21), the Natural Science Foundation of Hainan Province (2021RC169), the Hainan Provincial Key Research and Development Program (ZDYF2020177), and the Foundation of Hainan University [KYQD(ZR)1805].

## Conflict of interest

The authors declare that the research was conducted in the absence of any commercial or financial relationships that could be construed as a potential conflict of interest.

## Publisher’s note

All claims expressed in this article are solely those of the authors and do not necessarily represent those of their affiliated organizations, or those of the publisher, the editors and the reviewers. Any product that may be evaluated in this article, or claim that may be made by its manufacturer, is not guaranteed or endorsed by the publisher.

## Footnotes

^1^ https://www.r-project.org/
