# Supplementary material for: Adaptive changes of coral Galaxea fascicularis holobiont in response to nearshore stress
Source: Front Microbiol. 2022 Nov 8;13:1052776. doi: 10.3389/fmicb.2022.1052776 (PMC9678930; doi:10.3389/fmicb.2022.1052776)
Supplement: Supplementary file 1 [file Data_Sheet_1.docx]

**Supplementary Information:**

**Adaptive changes of coral *Galaxea fascicularis* holobiont in response to nearshore stress**

*Wentao Zhu ^a, c^**, Ming Zhu ^b, c^, Xiangbo Liu ^b, c^, Jingquan Xia ^c^, Rouwen Chen ^b^, Xiubao Li ^b, c*^*

^a^ College of Ecology and Environment, Hainan University, Haikou, China;

^b^ College of Marine Science, Hainan University, Haikou, China;

^c^ State Key Laboratory of Marine Resource Utilization in South China Sea, Hainan University, Haikou, China.

*Corresponding author: *Xiubao Li*

58 Renmin Road, Haikou, Hainan Province, 570228, China.

E-mail: [xiubaoli@hainanu.edu.cn](mailto:xiubaoli@hainanu.edu.cn)

**Supplementary Table 1**: Sampling information of investigated sites

| Station | lon | lat | Date | Depth(m) | Group |
| --- | --- | --- | --- | --- | --- |
| CJ1 | 108.8253 | 19.45103 | 2021/7/5 | 4 | HN |
| CJ2 | 108.8273 | 19.4519 | 2021/7/5 | 3 | HN |
| CJ3 | 108.8232 | 19.45145 | 2021/7/5 | 3 | HN |
| CJ4 | 108.7966 | 19.42305 | 2021/7/5 | 4 | HN |
| CJ5 | 108.7984 | 19.4244 | 2021/7/5 | 3 | HN |
| CJ6 | 108.7922 | 19.42195 | 2021/7/3 | 5 | HN |
| DCJ21 | 109.0862 | 19.67563 | 2021/7/3 | 3 | HN |
| DCJ22 | 109.089 | 19.66553 | 2021/7/3 | 3 | HN |
| DCJ23 | 109.1105 | 19.68355 | 2021/7/4 | 3 | HN |
| DCJ24 | 109.1064 | 19.62875 | 2021/7/4 | 4 | HN |
| DCJ25 | 109.1133 | 19.62918 | 2021/8/19 | 6 | HN |
| LC27 | 109.4677 | 19.9207 | 2021/8/19 | 7 | HN |
| M1 | 109.66 | 20.01023 | 2021/8/21 | 5 | HN |
| M2 | 109.6598 | 20.01028 | 2021/8/21 | 4 | HN |
| M3 | 109.6604 | 20.01037 | 2021/8/21 | 4 | HN |
| M4 | 109.6255 | 20.00272 | 2021/8/21 | 3 | HN |
| M5 | 109.6257 | 20.0035 | 2021/8/21 | 2 | HN |
| M6 | 109.6263 | 20.00417 | 2021/8/21 | 3 | HN |
| G4 | 112.2057 | 16.9575 | 2021/7/12 | 9 | XS |
| G7 | 112.2565 | 16.97 | 2021/7/14 | 7 | XS |
| G8 | 112.2585 | 16.9683 | 2021/7/14 | 6 | XS |
| G9 | 112.2698 | 16.9696 | 2021/7/14 | 8 | XS |
| G10 | 112.3055 | 16.9563 | 2021/7/13 | 10 | XS |
| G12 | 112.3061 | 16.9561 | 2021/7/13 | 6 | XS |
| G13 | 112.3071 | 16.9595 | 2021/7/13 | 6 | XS |
| G14 | 111.7504 | 16.4629 | 2021/7/17 | 9 | XS |
| G15 | 111.7416 | 16.4564 | 2021/7/17 | 8 | XS |
| G16 | 111.7362 | 16.4626 | 2021/7/18 | 4 | XS |
| G17 | 111.74 | 16.4695 | 2021/7/18 | 8 | XS |
| G19 | 111.5887 | 16.5014 | 2021/7/16 | 9 | XS |
| G20 | 111.5885 | 16.5086 | 2021/7/17 | 9 | XS |
| G21 | 111.5861 | 16.5107 | 2021/7/17 | 8 | XS |


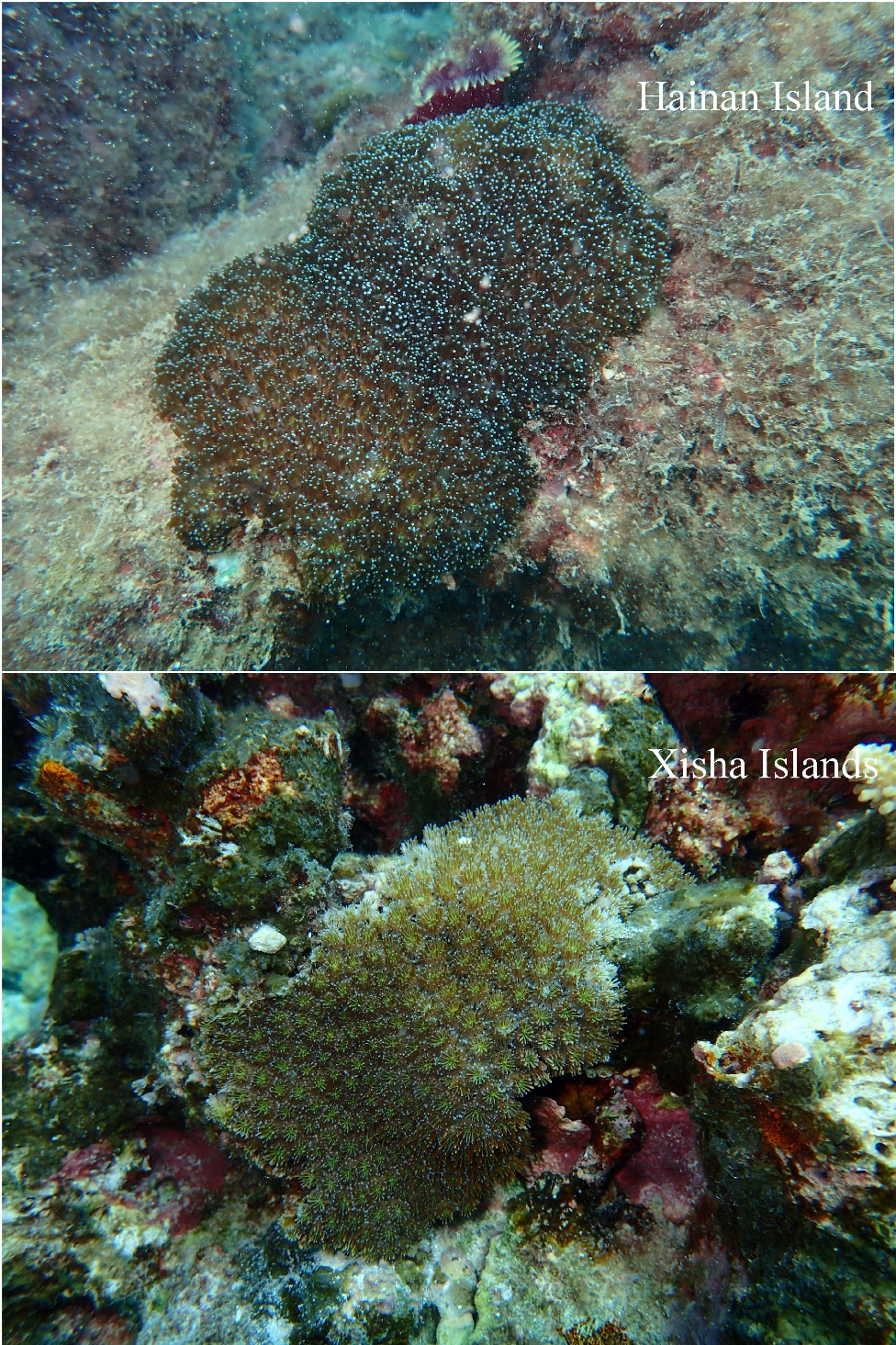


**Supplementary Figure 1**: The representative images of *Galaxea fascicularis* from Hainan Island and Xisha Islands.


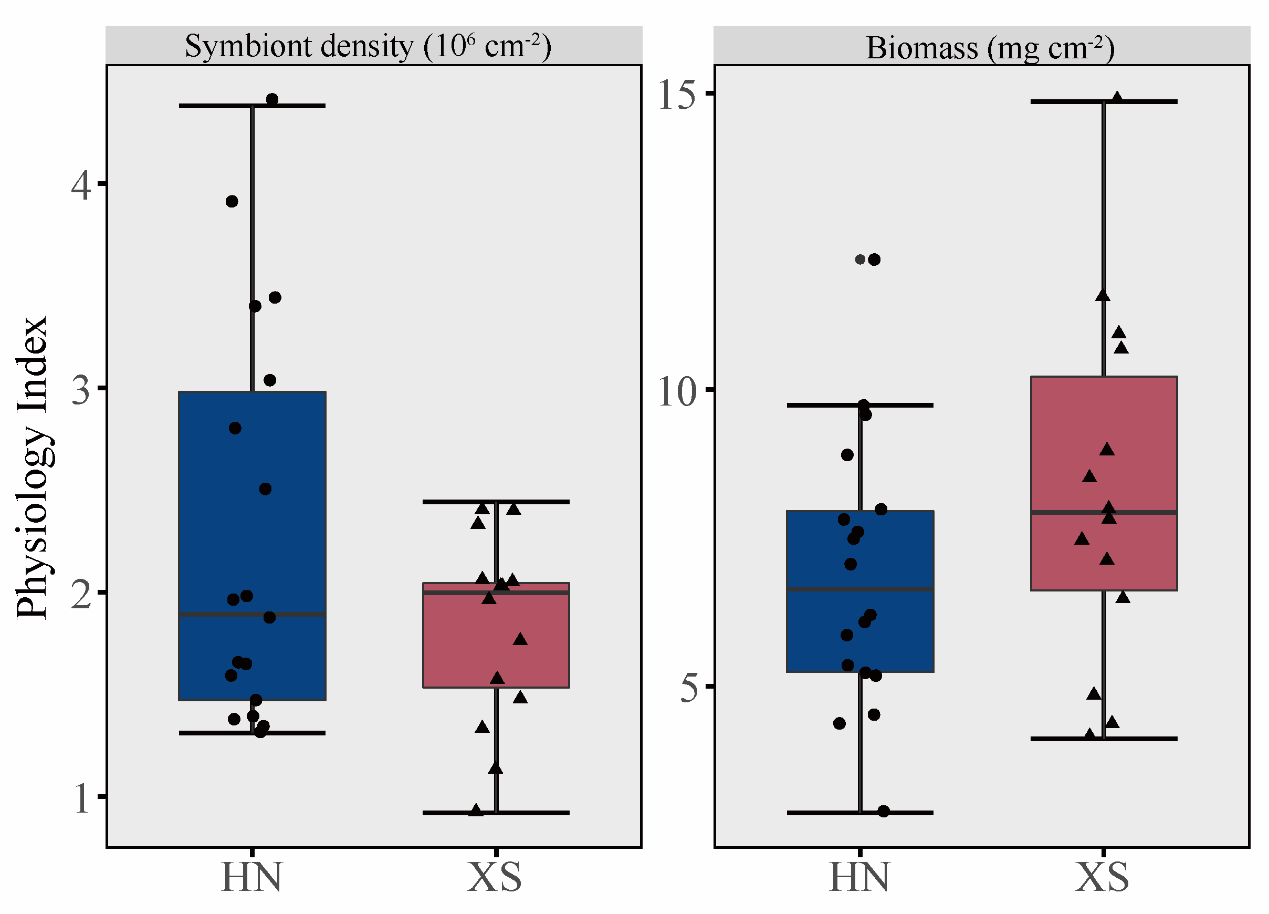


**Supplementary Figure 2**: Boxplots represent symbiont density and tissue biomass among sample replicates for sampling area. Center lines show medians, and whiskers indicate the full range of sample values.


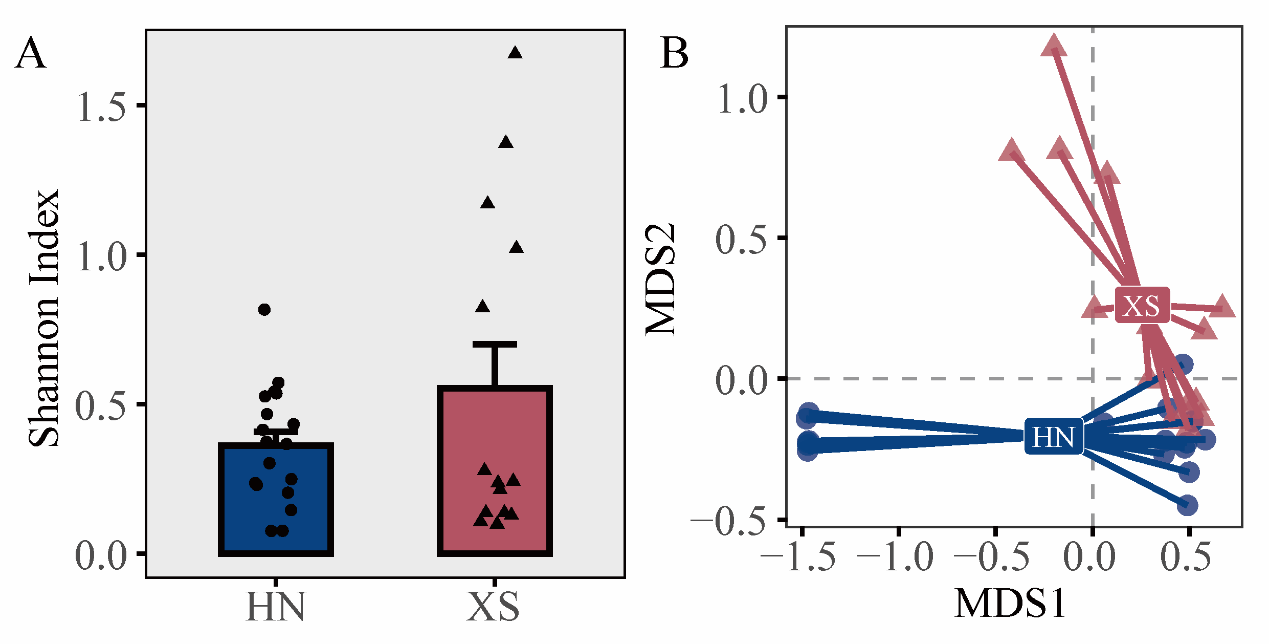


**Supplementary Figure 3**: Alpha diversity (based on the Shannon index) of the Symbiodiniaceae (A). NMDS plots based on the Bray–Curtis distance of Symbiodiniaceae community (B).


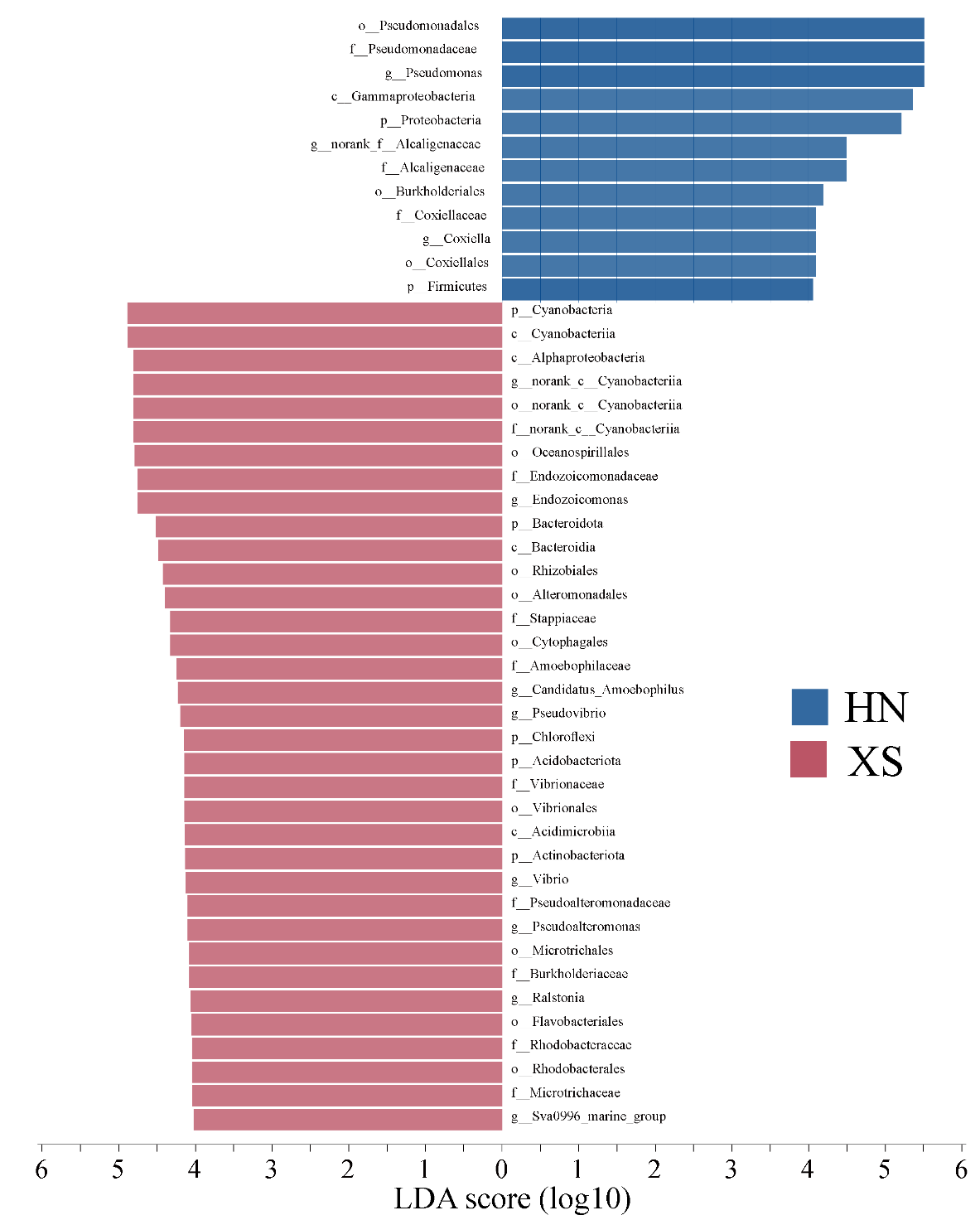


**Supplementary Figure 4**: Indicator microbial groups at each location using linear discriminant analysis.


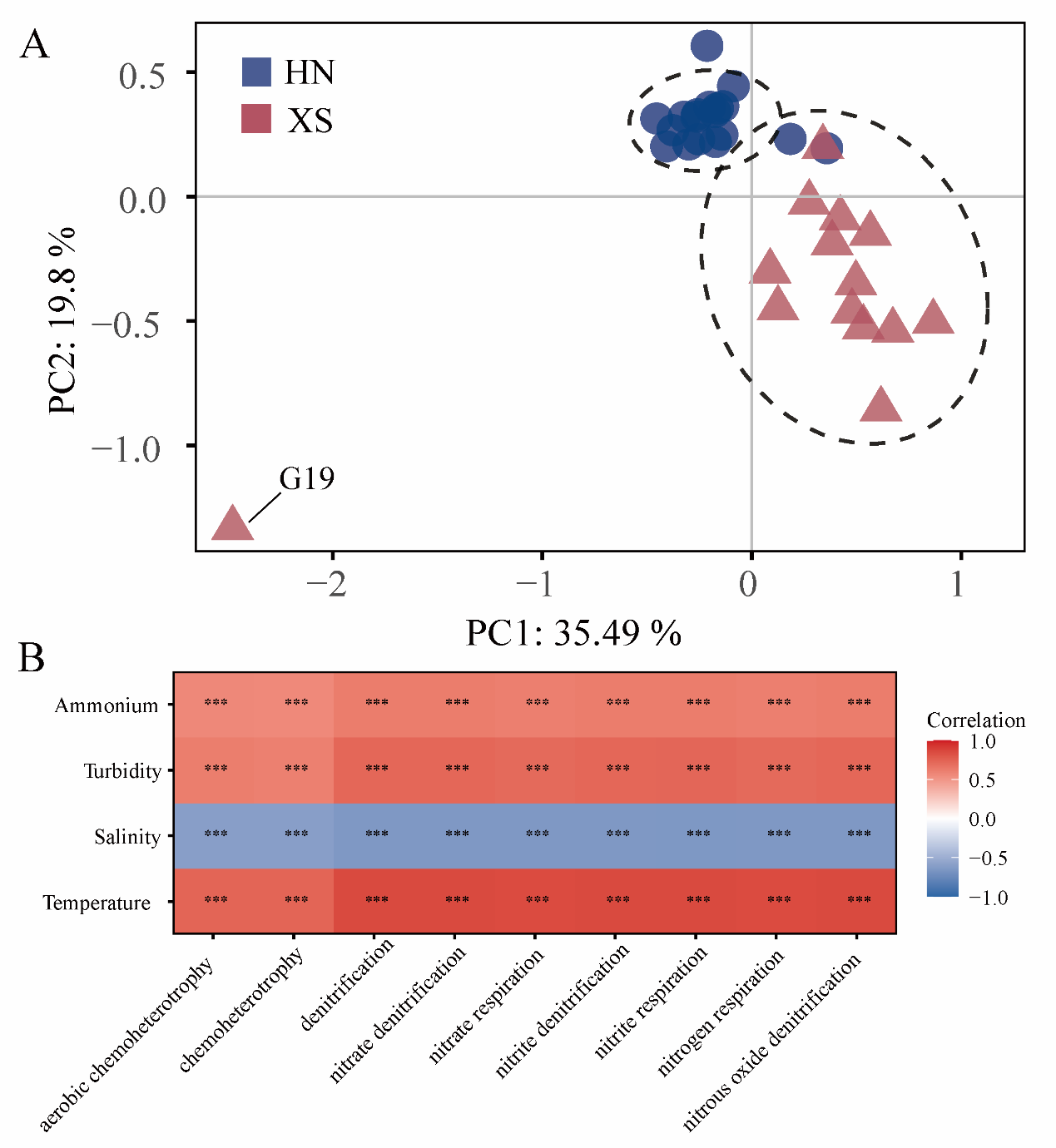


**Supplementary Figure 5**: PCA of the functional groups based on the FAPROTAX functional database (A). Heatmap of the correlations between the main functional groups and environmental indicators (B). A significant correlation was confirmed if the p-value was less than 0.001 (∗∗∗).


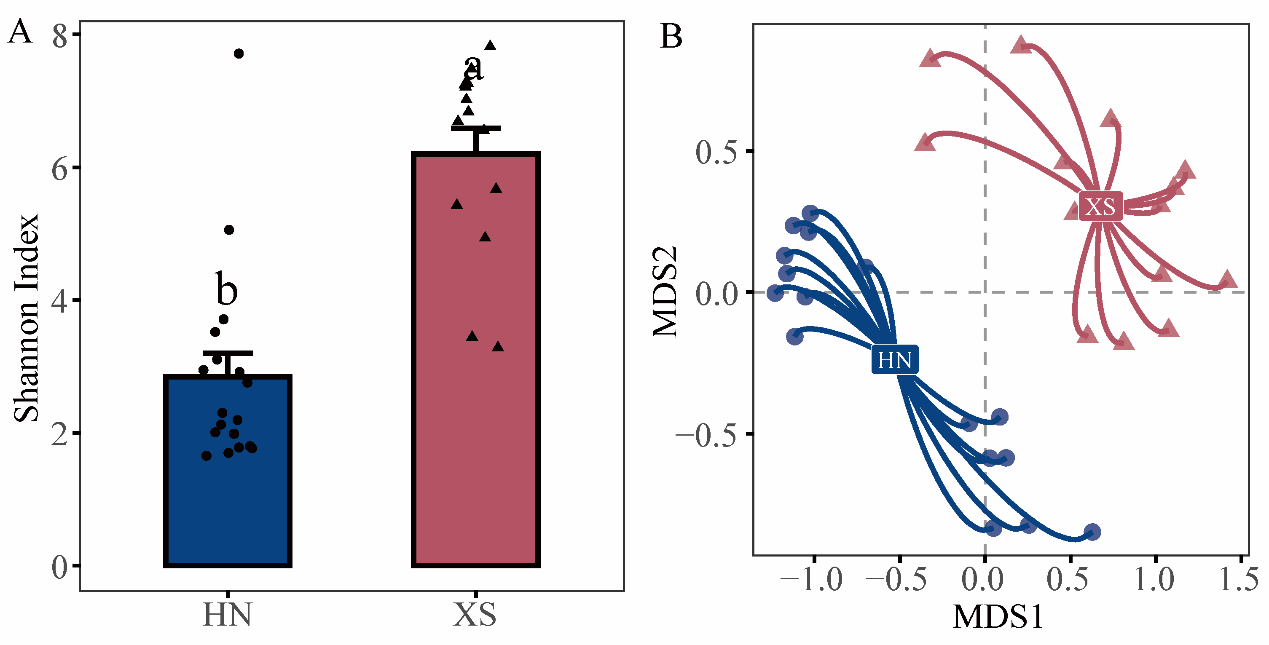


**Supplementary Figure 6**: Alpha diversity (based on the Shannon index) of the bacterial community (A). NMDS visualizations of beta diversity analysis using the Bray–Curtis metric separating samples by collection location (B).


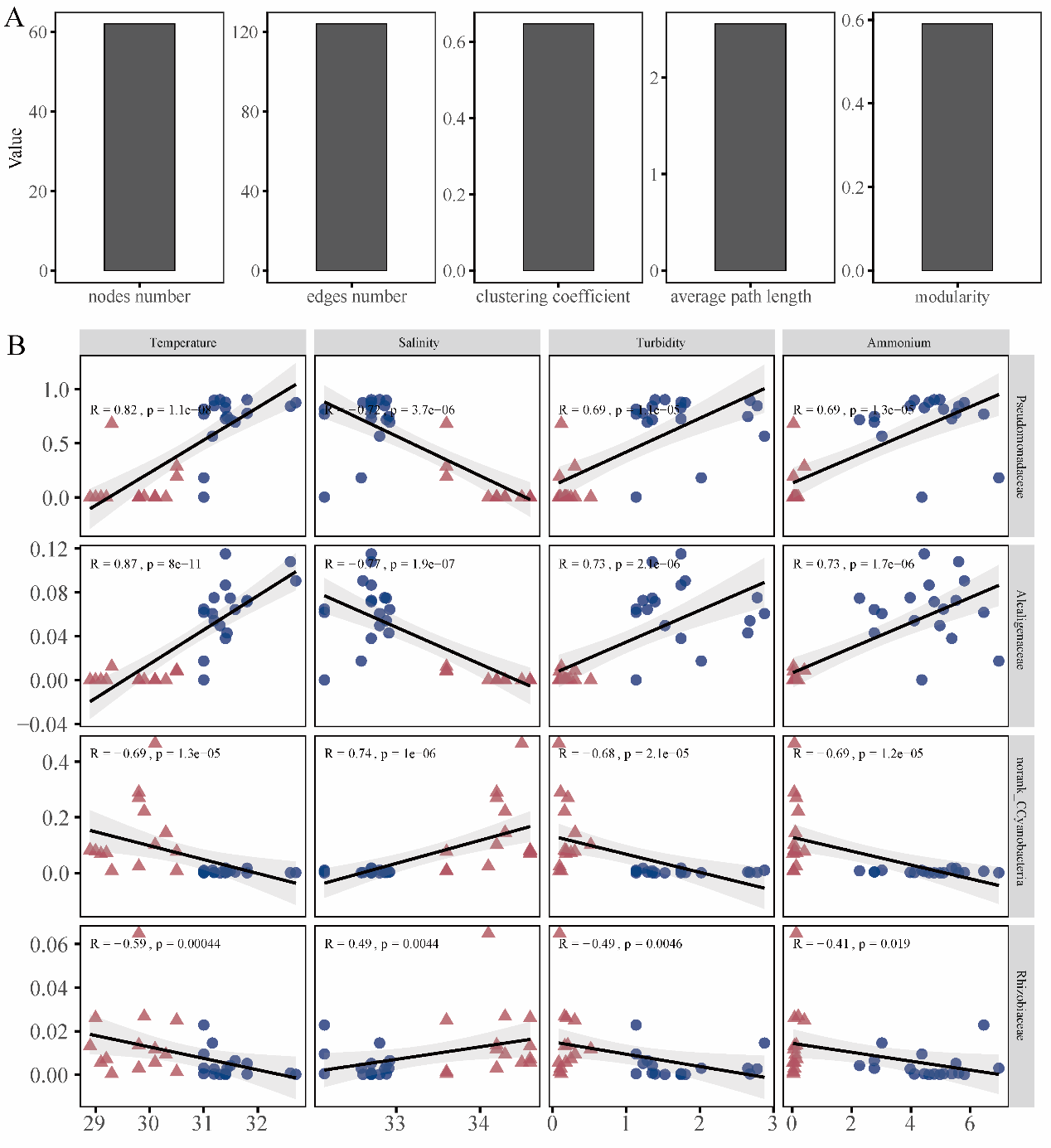


**Supplementary Figure 7**: Topological properties of the empirical networks of the bacterial community (A). Correlation between environmental parameters and relative abundance of key microbial taxa (B).
